# Biomedical insights into cell adhesion and migration—from a viewpoint of central nervous system tumor immunology

**DOI:** 10.3389/fcell.2015.00055

**Published:** 2015-10-14

**Authors:** Mitsugu Fujita, Takaaki Matsui, Akihiko Ito

**Affiliations:** ^1^Department of Microbiology, Faculty of Medicine, Kindai UniversityOsaka, Japan; ^2^Gene Regulation Research, Graduate School of Biological Sciences, Nara Institute of Science and TechnologyNara, Japan; ^3^Department of Pathology, Faculty of Medicine, Kindai UniversityOsaka, Japan

**Keywords:** cell adhesion, cell migration, cancer immunology, central nervous system, integrins, chemokines

## Fine-tuned orchestration of cell adhesion and migration

Cell adhesion and migration is a central process in the development and maintenance of multicellular organisms (Friedl et al., 2012). Tissue formation during embryonic development, wound healing, and immune responses all require the fine-tuned, orchestrated movement of cells. In this regard, recent cutting-edge studies have elucidated how cell adhesion and migration achieve three-dimensional collective cell movement (Rørth, 2012; Matsui et al., 2015). That is, collective cell movement uses a mechanical guidance system where each cell in the cell groups individually but coordinately guides the global motion of the cell groups. This mechanism is involved in a variety of biological reactions such as embryonic morphogenesis, organogenesis, immune reactions, and/or carcinogenesis (Friedl and Gilmour, 2009). In this review article, we will particularly pick up immune reactions in central nervous system (CNS) and CNS tumors to look into the fine-tuned mechanisms how cell adhesion and migration are involved in physiological condition as well as pathological conditions.

## Significance of cell adhesion in CNS/CNS tumor immunology

In the past, the CNS has been characterized as an immunologically privileged site. Nowadays, however, immune reactions are known to occur in the CNS (Ousman and Kubes, 2012). In particular, regarding immune cell trafficking to the brain, the mechanisms how leukocytes translocate from high velocity circulation into the brain parenchyma is explained as follows: (1) tethering/rolling, (2) activation, (3) adhesion, and (4) transmigration (Engelhardt, 2008). Briefly, leukocytes slow down on endothelial cells by selectin-mediated interactions expressed on both of the cell types. Then, at a reduced velocity, the leukocytes sense chemokines on the endothelial cells and become activated through G-protein signaling to upregulate integrins (Fujita et al., 2009). Finally, with this tight interaction in place, leukocytes transmigrate into the parenchyma to function. In the following section, we discuss major adhesion molecules that are relevant to this process.

### ICAM1-LFA1 interaction for T lymphocytes

ICAM1 (Intercellular Adhesion Molecule 1), which is also known as CD54, is a cell surface glycoprotein that is typically expressed on endothelial cells as well as distinct subsets of leukocytes (Rothlein et al., 1986). ICAM1 is a ligand for LFA1 (Lymphocyte Function-Associated molecule 1), a receptor primarily expressed on leukocytes. As mentioned above, when leukocytes slow down via interactions between LFA1 on leukocytes and ICAM1 on endothelial cells, the leukocytes become activated by chemokine signals (we will discuss later) and further upregulate the expression levels of the integrins. In particular, the interaction between ICAM1 and LFA1 is a crucial step in the generation of tumor-specific cytotoxic T lymphocytes (CTLs) (Jenkinson et al., 2005). It is also required for the *in situ* activation and the migration of the CTLs (Bachmann et al., 1997). Moreover, ICAM1 has been shown to play a pivotal role in physical and functional interaction between CNS tumors and CTLs (Ueda et al., 2009). Therefore, malignant CNS tumor cells reduce the expression levels of ICAM1 to achieve their tumor immune escape (Dunn et al., 2007).

### VCAM1-VLA4 interaction for T lymphocytes

VCAM1 (Vascular Cell Adhesion Molecule 1), which is also known as CD106, mediates the adhesion of lymphocytes, monocytes, eosinophils, and basophils to vascular endothelium (Cybulsky et al., 1991). It also functions in leukocyte-endothelial cell signal transduction. VCAM1 interacts with VLA4 (Very Late Antigen-4 or integrin α4β1) (Lin and Castro, 1998).

Expression of tissue-specific homing molecules directs antigen-experienced T lymphocytes to particular peripheral tissues. It is therefore essential to gain understanding of pivotal homing receptors that dictate CNS tumor-homing of T lymphocytes. It has been shown that *in vivo* imprinting of distinct homing phenotypes of T lymphocytes occurs in response to tumor-expressing antigens in intracerebral, subcutaneous, and intraperitoneal sites (Calzascia et al., 2005). In addition, CNS-homing type-1 CTLs (Tc1; the most potent effector T cells) but not its counterpart type-2 CTLs (Tc2) preferentially express VLA4 (Sasaki et al., 2007; Zhu et al., 2007). As mentioned above, ICAM1-LFA1 interaction enhances a stable interaction between VCAM1 and VLA4, which allows the cells to migrate into brain parenchyma (Sasaki et al., 2008).

### CADM1-mediated interaction for dendritic cells (DCs)

CADM1 (Cell Adhesion Molecule 1) is an immunoglobulin superfamily member that is expressed on neuron cells such as superior cervical ganglions (Watabe et al., 2003). In addition, a soluble form of CADM1 (sCADM1) can be generated as an alternative splicing variant, which is involved in directional neuron extension (Hagiyama et al., 2009). CADM1 has been shown to interact with an intracytoplasmic protein DAL1 (Yageta et al., 2002).

DCs are known to be antigen-presenting cells (APCs) of the mammalian immune system; they act as messengers between the innate and the adaptive immune systems (Palucka and Banchereau, 2012). Their main function is to process antigen material and present it on the cell surface to naive T lymphocytes to activate them. Therefore, they decide how T lymphocytes differentiate. Recent studies have shown that a certain subset of DCs express CADM1 (Dutertre et al., 2014). In addition, CADM1-postive and CADM1-negative subsets correspond to type-1 DCs (which induces type-1 T lymphocytes such as Tc1) and type-2 DCs, respectively. These data suggest that CNS tumors may preferentially induce immunosuppressive milieu by promoting CADM1-negative DCs in the tumor microenvironment.

## Significance of cell migration in CNS/CNS tumor immunology

In turn, regarding the cell migration in CNS immunology, the most important factors are chemokines because they act as a chemoattractant to guide the migration of leukocytes directly (Proudfoot, 2002). Chemokines are a family of cytokines and classified into four main subfamilies: CXC, CC, CX3C, and XC (Zlotnik and Yoshie, 2012). All of these proteins exert their biological effects by interacting with G protein-linked transmembrane receptors called chemokine receptors that are selectively found on the surfaces of their target cells. Some chemokines are involved in immune surveillance; they direct lymphocytes to the lymph nodes so that the lymphocytes can screen for invasion of pathogens by interacting with APCs residing in these tissues. Some chemokines have roles in development; they promote angiogenesis or guide cells to tissues that provide specific signals critical for cellular maturation. Other chemokines are inflammatory; they are released from a wide variety of cells in response to bacterial infection. Inflammatory chemokines function mainly as chemoattractants for leukocytes to recruit them from the blood to sites of infection or tissue damage. In the following sections, we discuss about the CNS tumor-relevant immunology from a viewpoint of chemokines.

### CCR7-CCL19/CCL21 axis for DCs

CCR7 is a chemokine receptor that is expressed by various subsets of leukocytes, and its ligands are CCL19 and CCL21 (Förster et al., 2008). These chemokines are constitutively expressed and control cell movement during homeostasis. CCR7-CCL19/21 chemokine axis is essentially involved in homing of activated DCs to the lymph nodes. Within lymph nodes of the systemic immune system, T lymphocytes establish close physical contacts with DCs, which allows their antigen-specific activation (Ganguly et al., 2013).

In contrast, in the CNS, a variety of cell populations have been postulated as primary APCs: vascular endothelial cells, smooth muscle cells, astrocytes, perivascular macrophages, choroid plexus epithelial cells, neurons, and DCs (Dunn et al., 2007). Presentation of CNS antigens by APCs can occur through multiple mechanisms (Walker et al., 2003): (1) APC uptake antigen within the CNS and migrate to lymph nodes to present antigens; (2) antigen drains to lymph nodes where APCs take them up to present; and (3) cells that express the antigen directly drain to lymph nodes and present their own antigen (direct presentation as opposed to cross presentation by DCs). In the process of lymph node-homing of DCs, CCR7 appears to play a central role in this process. Indeed, CCR7+ DCs injected in brain tumors have been shown to migrate to the cervical lymph nodes (CLNs) (Dunn et al., 2007; Fujita et al., 2009). Likewise, CCR7+ CD11c+ cells resembling classical bone marrow-derived DCs appear to be involved in other diseases such as CNS infectious and autoimmune encephalitis (Ganguly et al., 2013).

### CXCR3-CXCL10 axis for T lymphocytes

CXCR3 is a chemokine receptor that is rapidly induced on naïve T lymphocyte following activation and preferentially remains highly expressed on type-1 helper (Th1)-type CD4+ T lymphocytes, effector CD8+ T lymphocytes and innate-type lymphocytes such as natural killer (NK) and NKT cells (Groom and Luster, 2011). CXCR3 is activated by three interferon (IFN)-γ-inducible ligands CXCL9, CXCL10, and CXCL11. Among these chemokines, CXCL10 is induced by a variety of innate stimuli that induce IFN-α/β as well as the adaptive immune cell cytokine IFN-γ. CXCL10 has been attributed to several roles, such as (1) chemoattraction for monocytes/macrophages, T lymphocytes, NK cells, and dendritic cells; (2) promotion of T cell adhesion to endothelial cells; and (3) inhibition of bone marrow colony formation and angiogenesis.

In particular, Tc1 are known to efficiently traffic to the CNS tumor site through CXCR3-CCL10 axis compared with Tc2 (Nishimura et al., 2006; Zhu et al., 2007; Fujita et al., 2009). CXCR3 is uniquely up-regulated on Tc1, which is critical for efficient CNS tumor-homing of Tc1. Further information on mechanisms underlying efficient CNS-tumor homing of CTLs should be gained for development of truly effective immunotherapy strategies for CNS tumors.

### CCR2-CCL2 axis for macrophages/microglia

CCR2 is a chemokine receptor, which regulates the mobilization of monocytes from bone marrow to the inflammatory sites, and it has been extensively studied in CNS inflammation (Chu et al., 2014). CCR2 is activated by several chemokines, including CCL2, CCL7, CCL8, CCL12, CCL13, and CCL16. Activation of CCR2 results in directional migration of receptor-bearing cell types. Among them, CCL2 is known to be the most potent activator of CCR2 signaling, leading to monocyte transmigration. In the CNS tumor setting, CCL2 is secreted by tumor cells (Zhu et al., 2011). CCL2 can directly promote angiogenesis through the recruitment of tumor-associated macrophages (Salcedo et al., 2000). In addition, CCL2 has been shown to be critical for cell proliferation of CNS tumors, cancer cell metastasis, as well as tumor aggressiveness (Huang et al., 2007).

In the CNS, macrophages/microglial cells constitute the first line of cellular defense against a variety of stressors, participating in the regulation of innate and adaptive immune responses (Badie and Schartner, 2000). Many CNS tumors exhibit a prominent macrophage/microglia infiltrate. It is postulated that defense functions of macrophage/microglia against glioma are compromised in the tumor microenvironment. CNS macrophages/microglial cells expressed substantial levels of CCR2 and Toll-like receptors (TLRs), which are critical components for APCs to mediate innate immune responses to any infectious or traumatic challenge and activating adaptive immune responses. However, CNS macrophages/microglial cells do not appear to produce pro-inflammatory cytokines such as TNF-α, IL-1, or IL-6. Moreover, these cells, in addition to decreased surface expression of MHC class II (Watters et al., 2005), lack expression of the costimulatory molecules CD86, CD80, and CD40 critical for T cell activation, thereby unable to activate T cells properly *ex vivo* (Hussain et al., 2006). Therefore, in the CNS tumor microenvironment, macrophages/microglial cells are considered to be immunosuppressive so that they are potent therapeutic target for anti-CNS tumor immunotherapy (Fujita et al., 2010, 2011; Zhu et al., 2011).

### CCR4-CCL22 axis for regulatory T lymphocytes (tregs)

CD4+CD25+FoxP3+ Tregs are also found in the tumor microenvironment (Zou, 2006). There are four potential sources for Tregs in the CNS tumor microenvironment: the thymus, lymph nodes, bone marrow, and peripheral blood traffic to the tumor. Tregs express CCR4; abundant expression of CCL22, the ligand for CCR4, in the tumor microenvironment stimulates the tumor infiltration of Tregs. The tumor microenvironment contains molecules that can suppress APC differentiation and function. These dysfunctional APCs can in turn stimulate Treg differentiation. In addition, DCs can stimulate Treg expansion, and it is predicted that DCs in the tumor microenvironment move toward draining lymph nodes and further induce Treg expansion.

Similarly, the suppressive activity of Tregs is an important factor since they limit CTL-mediated destruction of CNS tumor cells. An increased ratio of CCR4+FoxP3+ Tregs to total CD4+ T cells correlates with impairment of CD4+ T cell proliferation in peripheral blood specimens obtained from CNS tumor patients (Fecci et al., 2006; Hussain et al., 2006). Moreover, Tregs are not present in normal brain tissue but are very rarely found in low-grade gliomas and oligodendrogliomas (Fujita et al., 2008; Heimberger et al., 2008). These studies also observed that Tregs infiltration differs significantly in the tumors according to lineage, pathology, and grade. In addition, treatment of CNS tumor-bearing hosts with anti-CD25 mAb delayed the tumor growth and prolonged the survival, suggesting that CCR4+CD4+CD25+ Tregs play an important role in suppressing the immune response to CNS tumors (El Andaloussi et al., 2006).

## Perspectives

We reviewed recent progress in the field of the CNS and CNS tumor immunology from a viewpoint of cell adhesion (primarily integrins) and migration (primarily chemokines). As discussed above, a line of studies have uncovered important roles of cell adhesion and migration during development of multicellular organisms as well as pathological conditions such as cancers. In addition, kinetics of cell adhesion and migration in the CNS provides a diverse scope for therapeutic strategies and target molecules. Moreover, it is clear that the CNS and CNS tumors are equipped with numerous layers of immunosuppression and immune escape mechanisms, perhaps including ones that we have not yet identified. These discoveries would allow us to develop strategies to overcome each of these mechanisms. The eventual success of tumor immunotherapies including those for CNS tumors will be dependent upon not only implementation of molecularly targeted trials that address multiple layers of challenges but also in-depth understanding of organ/tumor-specific immunology mediated by organ-specific cell adhesion and migration.

### Conflict of interest statement

The authors declare that the research was conducted in the absence of any commercial or financial relationships that could be construed as a potential conflict of interest.
